# Postoperative Adjuvant Treatment Strategy for Locally Advanced Rectal Cancer after Neoadjuvant Treatment

**DOI:** 10.1155/2021/8852699

**Published:** 2021-03-28

**Authors:** Jia-yi Li, Xuan-zhang Huang, Peng Gao, Xiao-wan Chen, Yong-xi Song, Xing-er Lv, Yv Fu, Qiong Xiao, Zhen-ning Wang

**Affiliations:** Department of Surgical Oncology and General Surgery, Key Laboratory of Precision Diagnosis and Treatment of Gastrointestinal Tumors, Ministry of Education, The First Affiliated Hospital of China Medical University, 155 North Nanjing Street, Heping District, Shenyang City 110001, China

## Abstract

**Background:**

Neoadjuvant (chemo) radiotherapy is used as a standard treatment for locally advanced rectal cancer (LARC), but there is no general consensus on either the efficacy of postoperative adjuvant chemotherapy in patients with LARC after neoadjuvant treatment and surgery, or whether the addition of oxaliplatin to adjuvant chemotherapy provides survival benefits.

**Methods:**

We performed a meta-analysis of data from the PubMed and Embase databases. We included patients with LARC who received neoadjuvant (chemo) radiotherapy and curative surgery. Overall survival (OS), disease-free survival (DFS), toxicity, and compliance were analyzed in the oxaliplatin/fluorouracil- (OX/FU-) based group compared with the FU-based group, and in the chemotherapy group compared with the observation group.

**Results:**

Twenty studies were included in the analysis. Our results indicated that adjuvant chemotherapy prolonged OS (hazard ratio [HR] = 0.78, 95%CI = 0.67–0.91) in patients with LARC treated with neoadjuvant (chemo) radiotherapy and surgery compared with those in the observation group. Subgroup analysis showed the same results in both the ypStage II and ypStage III groups. Compared with those in the observation group, patients in the chemotherapy group also showed an increase in DFS (HR = 0.75, 95%CI = 0.60–0.93). No significant increase was observed in OS (HR = 1.04, 95%CI = 0.87–1.24) or DFS (HR = 0.98, 95%CI = 0.76–1.27) when oxaliplatin was added to FU-based adjuvant chemotherapy, as compared with the FU-based treatment, and subgroup analysis also indicated no survival benefits in the clinical stage II, clinical stage III, ypStage II, and ypStage III groups.

**Conclusions:**

For patients with LARC who have already received neoadjuvant (chemo) radiotherapy and curative surgery, adjuvant chemotherapy improves OS over that in the observation group. Adding oxaliplatin to FU-based adjuvant chemotherapy does not confer survival benefits beyond those from FU-based adjuvant chemotherapy.

## 1. Introduction

Colorectal cancer is the second most frequent cancer in women and the third most frequent cancer in men [1]. To date, surgical resection is the main radical treatment. According to the NCCN guidelines, the standard treatment is postoperative adjuvant chemotherapy with or without oxaliplatin for patients with locally advanced rectal cancer (LARC) after neoadjuvant treatment [2]. However, according to the ESMO guidelines, the available evidence is insufficient for the use of postoperative chemotherapy after neoadjuvant chemoradiation [3]. Moreover, a consensus has not been reached on whether adjuvant chemotherapy should be used, and which specific chemotherapy regimens can be used after neoadjuvant treatment. Although some studies have demonstrated that adjuvant chemotherapy should be used for patients with LARC after neoadjuvant chemoradiation and surgery [4–8], the effects of adjuvant chemotherapy are unclear in patients with LARC receiving neoadjuvant chemoradiation.

An oxaliplatin-based adjuvant chemotherapy regimen for LARC patients who have received neoadjuvant treatment has been mainly extrapolated from results achieved with oxaliplatin-containing adjuvant chemotherapy in lymph node-positive colon cancer patients [9, 10], a strategy that is also considered as an optional adjuvant chemotherapy for rectal cancer in the NCCN guidelines. However, whether oxaliplatin meaningfully affects the survival outcomes in patients with rectal cancer after neoadjuvant treatment remains unclear. In the German CAO/ARO/AIO-04 trial, the addition of oxaliplatin to adjuvant chemotherapy has been found to be beneficial in terms of disease-free survival (DFS) in patients with LARC after neoadjuvant chemoradiation with oxaliplatin as an additional radiosensitizer [11]. In the ADORE trial, oxaliplatin-based adjuvant chemotherapy was found to significantly increase DFS but not overall survival (OS) in patients with ypStage III rectal cancer [12]. However, the final results of the PETACC-6 trial, which added oxaliplatin to both neoadjuvant chemoradiation (as an radiosensitizer) and adjuvant chemotherapy, have indicated no survival benefits in patients with LARC receiving oxaliplatin/fluorouracil-based (OX/FU-based) adjuvant chemotherapy compared with FU-based adjuvant chemotherapy [13]. In addition, oxaliplatin inevitably results in toxicity, thus, leading to poor compliance in postoperative chemotherapy. In a random trial, more than 40% of the safety population who received neoadjuvant chemoradiation and subsequently at least one cycle of oxaliplatin-based adjuvant chemotherapy had grade 3/4 toxicity, and only 48.1% of patients completed all the planned cycles [14]. Therefore, the use of oxaliplatin should be comprehensively considered in terms of all aspects including efficacy, toxicity, and compliance. Regarding whether adjuvant chemotherapy should be used, although one study has reported that adjuvant chemotherapy improves OS among patients treated with neoadjuvant chemoradiation [15], some studies have found that adjuvant chemotherapy does not improve OS or DFS in patients with LARC [16]. Some trials have also indicated that adjuvant chemotherapy may be beneficial in ypStage III patients only but not in ypStage II LARC patients [12, 17]. Furthermore, for patients with rectal cancer downstaged to ypStage 0 or I after neoadjuvant chemoradiation, the survival benefits of adjuvant chemotherapy also remain unclear, although adjuvant chemotherapy is typically considered. Some studies have shown that adjuvant chemotherapy confers survival benefits to patients with ypStage 0 and ypT2N0M0 (ypStage I) [15, 18]; however, other studies have reported that adjuvant chemotherapy is not necessary for patients with rectal cancer achieving either ypStage 0 or ypStage I [19, 20]. Therefore, it is of great significance in clinical practice to explore whether the survival benefits of adjuvant chemotherapy are related to pathologic tumor stage after neoadjuvant treatment in rectal cancer.

In this meta-analysis, we sought to summarize and further assess the survival benefits of postoperative chemotherapy following neoadjuvant treatment and curative surgery for patients with LARC and examine the role of oxaliplatin in adjuvant chemotherapy.

## 2. Materials and Methods

This meta-analysis was conducted in accordance with the Preferred Reporting Items for Systematic Reviews and Meta-Analyses (PRISMA) Guidelines (Supplementary File 1) [21], and the Newcastle–Ottawa Scale (NOS) criteria were used to assess the methodological quality of the included studies [22].

### 2.1. Search Strategy

We performed an integrated search in PubMed and Embase until December 2019 using MeSH/main keywords of “neoadjuvant chemotherapy,” “neoadjuvant radiotherapy,” “neoadjuvant chemoradiotherapy,” “neoadjuvant treatment,” “neoadjuvant treatments,” “neoadjuvant therapy,” “neoadjuvant therapies,” “preoperative chemotherapy,” “preoperative radiotherapy,” “preoperative chemoradiotherapy,” “preoperative treatment,” “preoperative treatments,” “preoperative therapy,” “preoperative therapies,” “pre-operative chemotherapy,” “pre-operative radiotherapy,” “pre-operative chemoradiotherapy,” “pre-operative treatment,” “pre-operative treatments,” “pre-operative therapy,” “pre-operative therapies”, “rectal cancer,” “colorectal cancer,” and “oxaliplatin.” When multiple articles using the same patient population with the same endpoints were found, the most informative one was chosen for inclusion in the study. We also reviewed the references for the obtained studies to avoid missing relevant studies. The detailed search strategies and search results are shown in Supplementary File 2.

### 2.2. Eligibility Criteria

The eligible studies met the following inclusion criteria: (i) patients were diagnosed with rectal cancer; (ii) all patients received preoperative (chemo) radiotherapy; (iii) all patients underwent surgical resection of rectal cancer; (iv) studies compared chemotherapy with observation or compared postoperative OX/FU-based chemotherapy with FU-based chemotherapy (OX/FU-based chemotherapy included 5-FU and leucovorin plus oxaliplatin [FOLFOX] or capecitabine plus oxaliplatin [CAPEOX] and FU-based chemotherapy included 5-FU alone or capecitabine alone, because capecitabine and 5-FU are homologous chemotherapeutic drugs and are recommended adjuvant chemotherapeutic drugs for LARC patients after neoadjuvant treatment); and (v) the outcomes of studies included OS, DFS, toxicity, or compliance. Studies were excluded according to the following criteria: (i) studies of patients diagnosed with diseases other than rectal cancer; (ii) studies of patients who did not receive preoperative (chemo) radiotherapy; (iii) studies that compared preoperative OX/FU-based therapy with preoperative FU-based therapy; (iv) reviews, meta-analyses, and case reports; and (v) studies without outcomes relevant to this analysis. Two reviewers assessed all studies independently, and the final selected studies were determined on the basis of agreement between the two reviewers.

### 2.3. Data Extraction

Two researchers completed the extraction of suitable data after reviewing the full text of included studies, and all disagreements were settled by discussion. The detailed information extracted from studies included author, year of publication, country, study design, number of patients, age, follow-up duration, TNM stage, tumor location from anal verge, neoadjuvant treatment regimen, adjuvant treatment regimen, type of surgery, median interval from surgery to adjuvant chemotherapy, and study outcomes including OS, DFS, compliance (completion of planned number of cycles), and toxicity (e.g., vomiting, nausea, neuropathy, allergic reaction, diarrhea, and hand-foot syndrome).

### 2.4. Statistical Analysis

The primary endpoints were DFS and OS. The secondary endpoints were compliance and toxicity. We evaluated the primary endpoint with the hazard ratio (HR) and 95% confidence interval (CI). Then, we calculated the pooled risk ratio (RR) with 95% CI to assess toxicity. If a study did not provide the HR or 95% CI directly, we used published data to obtain the statistics by using the methods reported by Tierney et al. [23].

We analyzed the overall OS and DFS for the OX/FU-based group versus the FU-based group, and the chemotherapy group versus the observation group. Additionally, we conducted subgroup analysis according to study design, pathologic tumor stage after neoadjuvant treatment (ypTNM), preoperative clinical tumor stage (cTNM), and neoadjuvant treatment regimen. Statistical analysis of compliance and toxicity was performed on the basis of data from the studies. According to the heterogeneity, we used a random-effects model when *I*^2^ > 50% or *p* < 0.1; otherwise, we used a fixed-effects model [24]. Publication bias was assessed with funnel plots with Begg's and Egger's tests [25, 26]. Findings were considered significant with a two-sided *p* value ≤ 0.05.

All analyses were performed in Stata software, version 12.0 (2011; Stata Corp., College Station, TX, USA).

## 3. Results

### 3.1. Study Research

**Figure 1:**
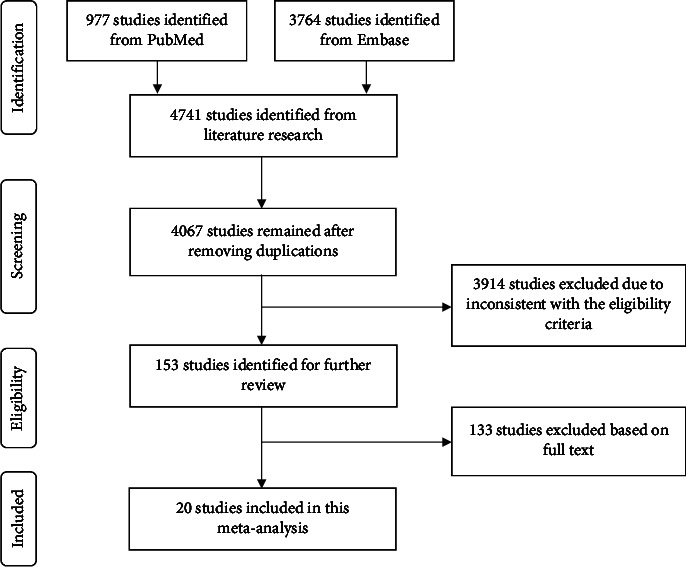
Literature search and study selection.

A total of 4741 studies were retrieved by the electronic search in total (977 studies were from PubMed, and 3764 studies were from Embase). A total of 4067 studies remained after the elimination of duplicates. Subsequently, 3914 articles were removed on the basis of the eligibility criteria according to the titles and abstracts, after which 153 articles were retained. After further reading and evaluation of the full text, 20 studies were ultimately included in this meta-analysis, including seven randomized controlled trials (RCTs) and 13 non-RCTs (nRCTs) [4–6, 8, 11–16, 27–36]. The detailed research steps are shown in Figure 1.

### 3.2. Characteristics of Included Studies

Twenty studies on 30662 patients were enrolled in the meta-analysis. Among the included studies, eight were from Europe, four from the USA, three from China, three from Korea, one from Canada, and one from Israel. Regarding the interventions, four studies had two arms containing an OX/FU-based group and FU-based group, 13 studies contained a chemotherapy group and observation group, and the other three studies contained an OX/FU-based group, FU-based group, and observation group. In terms of the neoadjuvant treatment regimen, preoperative long-course radiation with chemotherapy was conducted in 18 studies, long-course radiation alone or with chemotherapy was conducted in one study, and short-course radiation alone or with chemotherapy was used in one study. The follow-up duration of most studies was more than 3 years, four studies had longer durations of more than 5 years, and only two studies had durations of less than 1 year. The detailed baseline characteristics and study quality of the included studies are listed in Table 1.

### 3.3. Disease-Free Survival

**Figure 2:**
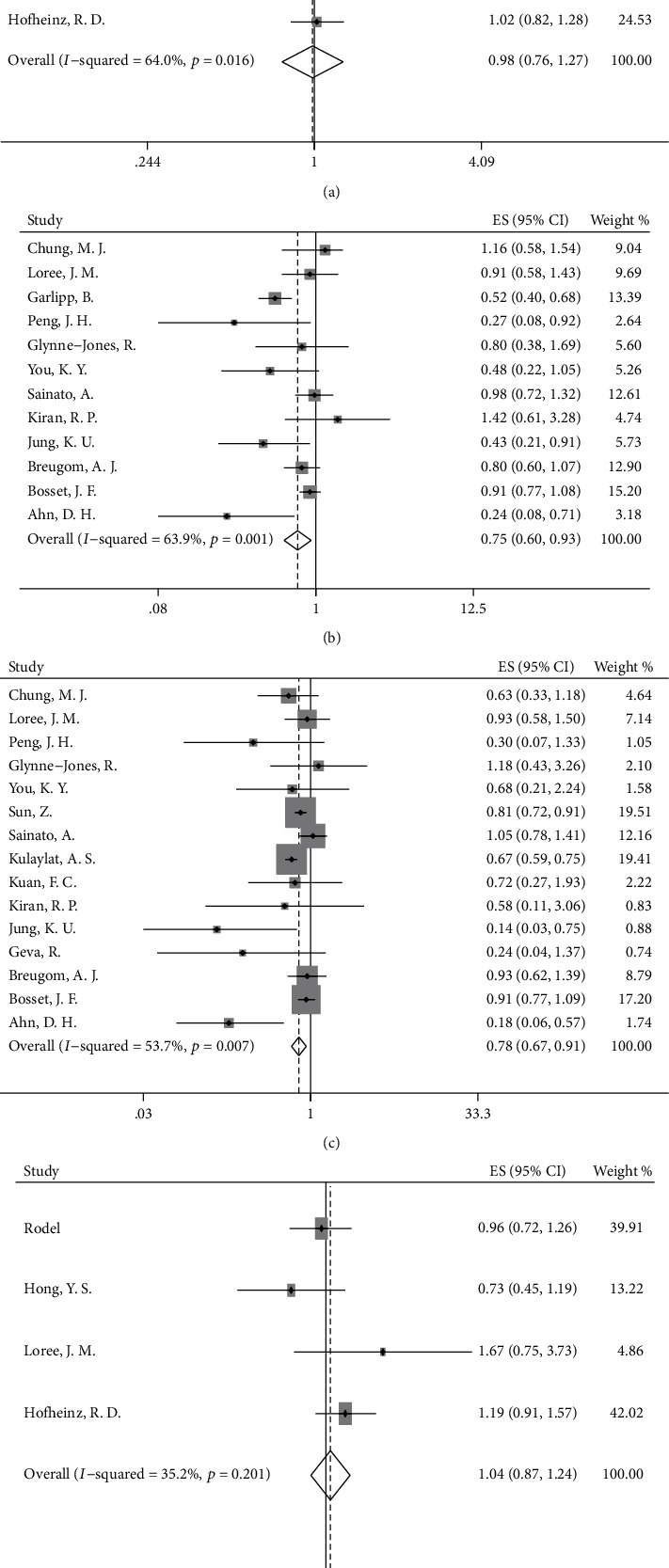
Forest plot based on survival outcomes. (a) Disease-free survival (DFS) in oxaliplatin/fluorouracil- (OX/FU-) based group versus fluorouracil- (FU-) based group; (b) DFS in chemotherapy group versus observation group; (c) overall survival (OS) in chemotherapy group versus observation group; (d) OS in OX/FU-based group versus FU-based group.

**Figure 3:**
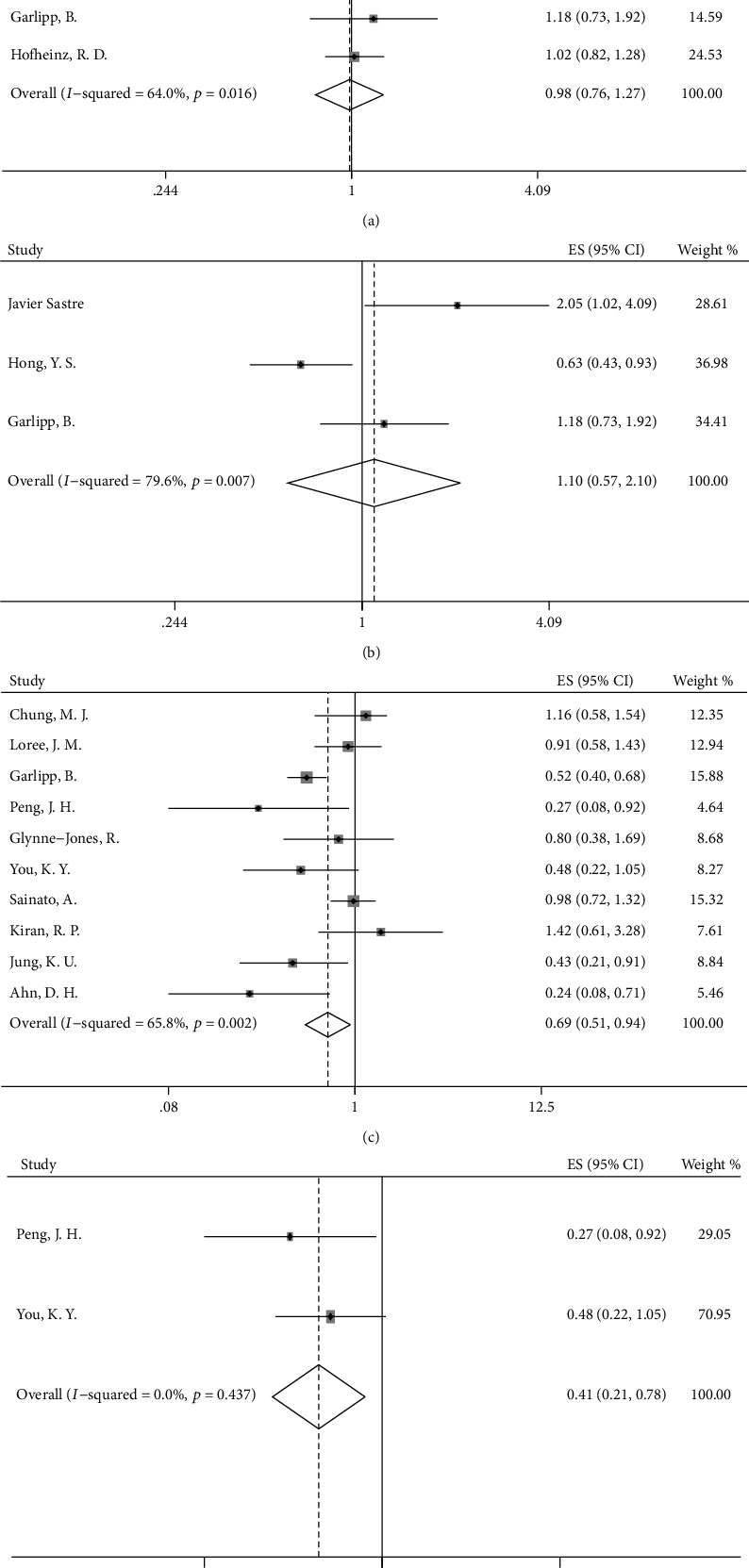
Forest plot based on disease-free survival after neoadjuvant chemoradiation. (a) OX/FU-based group versus FU-based group; (b) OX/FU-based group versus FU-based group after preoperative FU-based chemoradiation; (c) chemotherapy group versus observation group; (d) chemotherapy group versus observation group after preoperative OX/FU-based chemoradiation.

**Figure 4:**
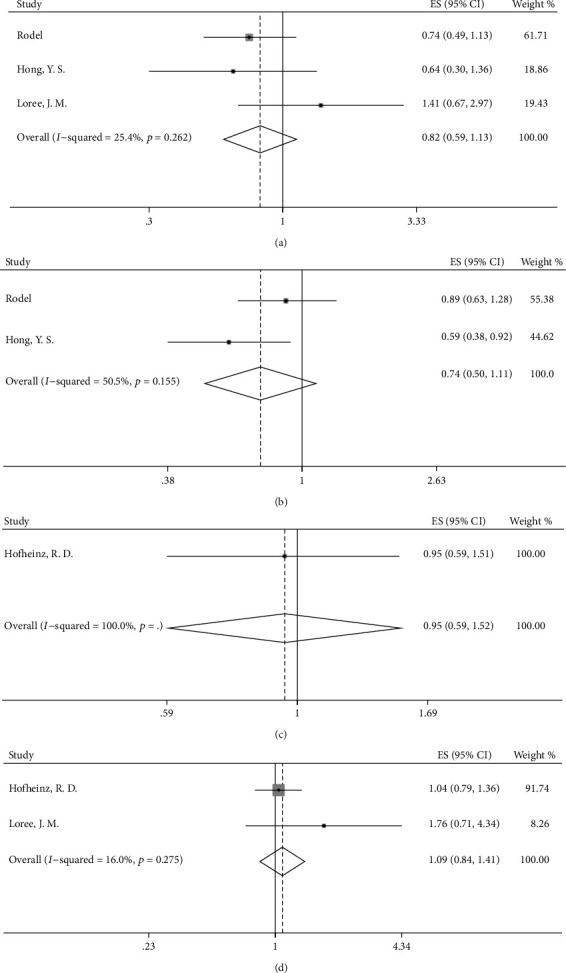
Forest plot based on disease-free survival and tumor stage. (a) DFS in OX/FU-based group versus FU-based group in patients with ypStage II; (b) DFS in OX/FU-based group versus FU-based group in patients with ypStage III; (c) DFS in OX/FU-based group versus FU-based group in patients with clinical stage II; (d) DFS in OX/FU-based group versus FU-based group in patients with clinical stage III.

According to our analyses, DFS did not significantly differ in the OX/FU-based group and the FU-based group (HR = 0.98, 95%CI = 0.76–1.27, *p* = 0.906, Figure 2(a)). To clarify the effect of the neoadjuvant treatment regimen on survival outcomes, we conducted a subgroup analysis based on the neoadjuvant treatment regimen to explore the differences in survival between the OX/FU-based group and FU-based group. After elimination of the patients treated with only preoperative radiation without preoperative chemotherapy, the DFS was not increased when oxaliplatin was added to FU-based adjuvant chemotherapy after long-course neoadjuvant chemoradiation (HR = 0.98, 95%CI = 0.76–1.27, *p* = 0.906, Figure 3(a)). In addition, we found no significant increase in DFS in the OX/FU-based group versus the FU-based group when preoperative FU-based chemotherapy was used as a radiosensitizer in the long-course chemoradiation (HR = 1.10, 95%CI = 0.57–2.10, *p* = 0.782, Figure 3(b)). However, we were unable to evaluate the survival benefits in the OX/FU-based group versus the FU-based group in the patients treated with preoperative short-course radiotherapy alone or long-course chemoradiation with preoperative OX/FU-based chemotherapy as a radiosensitizer, owing to insufficient data. Moreover, the subgroup analysis based on ypN indicated no difference in DFS between the OX/FU-based group and FU-based group in both ypN^−^ and ypN^+^ patients (ypN0: HR = 0.83, 95%CI = 0.64–1.08, *p* = 0.160; ypN1: HR = 1.05, 95%CI = 0.52–2.10, *p* = 0.897; ypN2: HR = 0.75, 95%CI = 0.33–1.70, *p* = 0.492). To investigate the specific patient group that would benefit from adding oxaliplatin to adjuvant chemotherapy, we further conducted a subgroup analysis based on ypStage. The OX/FU-based group did not show an increase in DFS over that in the FU-based group in either the ypStage II or ypStage III groups (ypStage II: HR = 0.82, 95%CI = 0.59–1.13, *p* = 0.225, Figure 4(a); ypStage III: HR = 0.74, 95%CI = 0.50–1.11, *p* = 0.142, Figure 4(b)). However, we could not evaluate the survival benefits of OX/FU-based versus FU-based adjuvant chemotherapy in ypStage I or ypStage 0, owing to insufficient data. In addition to pathologic tumor stage, the clinical tumor stage is an important factor used to define the guidelines for recommendation of administration of adjuvant chemotherapy; thus, we also conducted further subgroup analysis based on the clinical tumor stage, and the results also showed that DFS was not significantly different between the OX/FU-based group and FU-based group in both clinical stage II and III subpopulation (clinical stage II: HR = 0.95, 95%CI = 0.59–1.52, *p* = 0.831, Figure 4(c); clinical stage III: HR = 1.09, 95%CI = 0.84–1.41, *p* = 0.533, Figure 4(d)).

Patients treated with adjuvant chemotherapy showed improved DFS over that in the observation group (HR = 0.75, 95%CI = 0.60–0.93, *p* = 0.008, Figure 2(b)), and subgroup analysis based on the neoadjuvant treatment regimen showed similar results in the subgroup of patients receiving preoperative long-course chemoradiation (HR = 0.69, 95%CI = 0.51–0.94, *p* = 0.018, Figure 3(c)). Furthermore, a longer DFS was observed in patients receiving chemotherapy than in patients in the observation group when patients were treated with preoperative radiotherapy and preoperative OX/FU-based chemotherapy as a radiosensitizer (HR = 0.41, 95%CI = 0.21–0.78, *p* = 0.007, Figure 3(d)). Although preoperative neoadjuvant radiotherapy may affect the survival benefits of adjuvant treatment in patients with rectal cancer, there were insufficient data to conduct a corresponding subgroup analysis. In an RCT of 473 rectal cancer patients, 86% of the patients who received preoperative short-course radiotherapy and 14% of patients who received preoperative long-course chemoradiation were randomly assigned to a chemotherapy group and observation group, and the results showed a similar 5-year DFS in the chemotherapy group versus the observation group (62.7% versus 55.4%) [35]. However, there were no sufficient data to support other subgroup analyses. The detailed results are shown in Table 2.

### 3.4. Overall Survival

**Figure 5:**
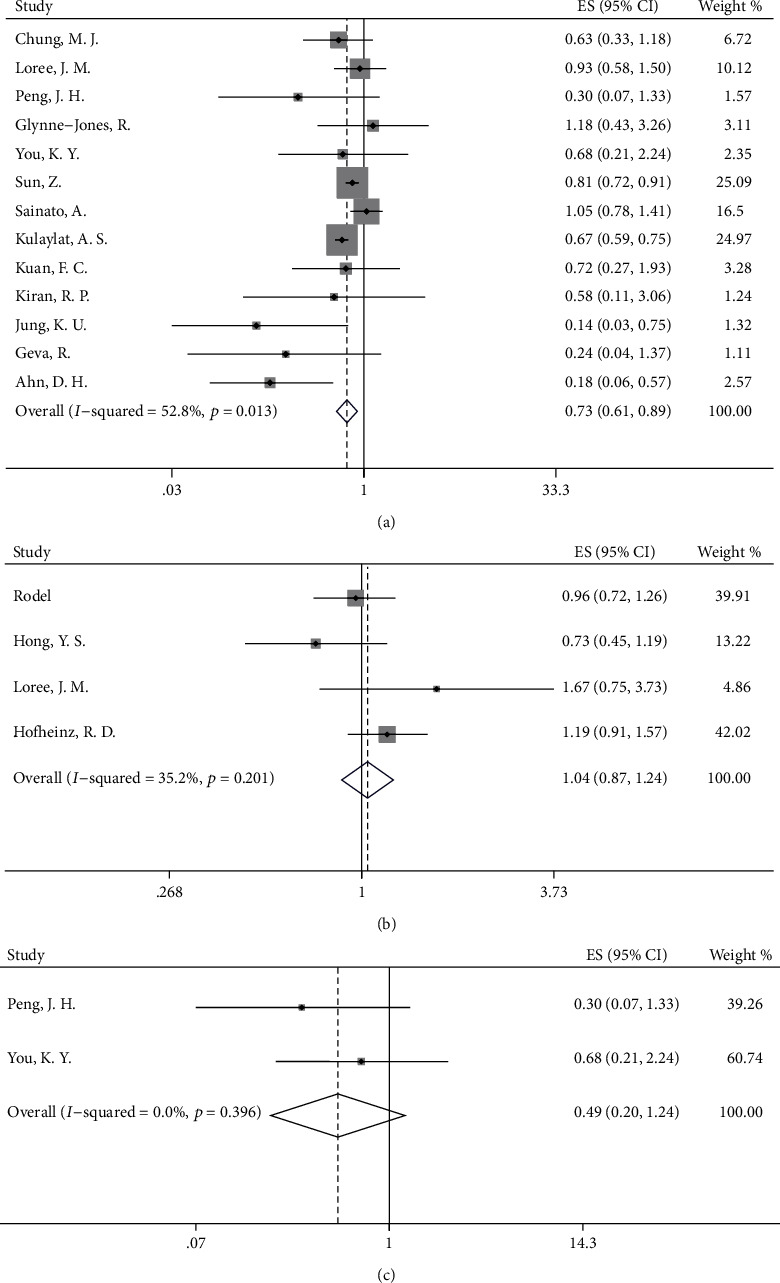
Forest plot based on overall survival after neoadjuvant chemoradiation. (a) Chemotherapy group versus observation group; (b) OX/FU-based group versus FU-based group; (c) chemotherapy group versus observation group after preoperative OX/FU-based chemoradiation.

Our results indicated a significantly increased OS in patients receiving chemotherapy (HR = 0.78, 95%CI = 0.67–0.91, *p* = 0.002, Figure 2(c)) compared with individuals in the observation group. However, a clear difference in OS was not found between the OX/FU-based group and FU-based group (HR = 1.04, 95%CI = 0.87–1.24, *p* = 0.656, Figure 2(d)). Subgroup analyses based on long-course neoadjuvant chemoradiation strategy showed similar results to the overall analysis. Increased OS was observed in the chemotherapy group versus the observation group (HR = 0.73, 95%CI = 0.61–0.89, *p* = 0.001, Figure 5(a)), and adding oxaliplatin to FU-based adjuvant chemotherapy was not beneficial for OS, as compared with that in the FU-based group (HR = 1.04, 95%CI = 0.87–1.24, *p* = 0.656, Figure 5(b)). Moreover, a tendency toward increased OS was found in the comparison between the chemotherapy group and observation group for patients receiving preoperative radiotherapy and preoperative OX/FU-based chemotherapy as a radiosensitizer (HR = 0.49, 95%CI = 0.20–1.24, *p* = 0.133, Figure 5(c)). However, the effects of different neoadjuvant chemotherapy strategies on survival benefits could not be evaluated in the comparison between the OX/FU-based group and FU-based group, owing to insufficient data. For preoperative short-course radiotherapy, in the RCT in which most rectal cancer patients (86% patients) treated with preoperative short-course radiotherapy alone, the survival rates in the adjuvant chemotherapy group and observation group were similar (80.4% vs. 79.2%), and no significant increase in OS was observed with adjuvant chemotherapy compared with observation (HR = 0.93, 95%CI = 0.62–1.39, *p* = 0.73) [35].

**Figure 6:**
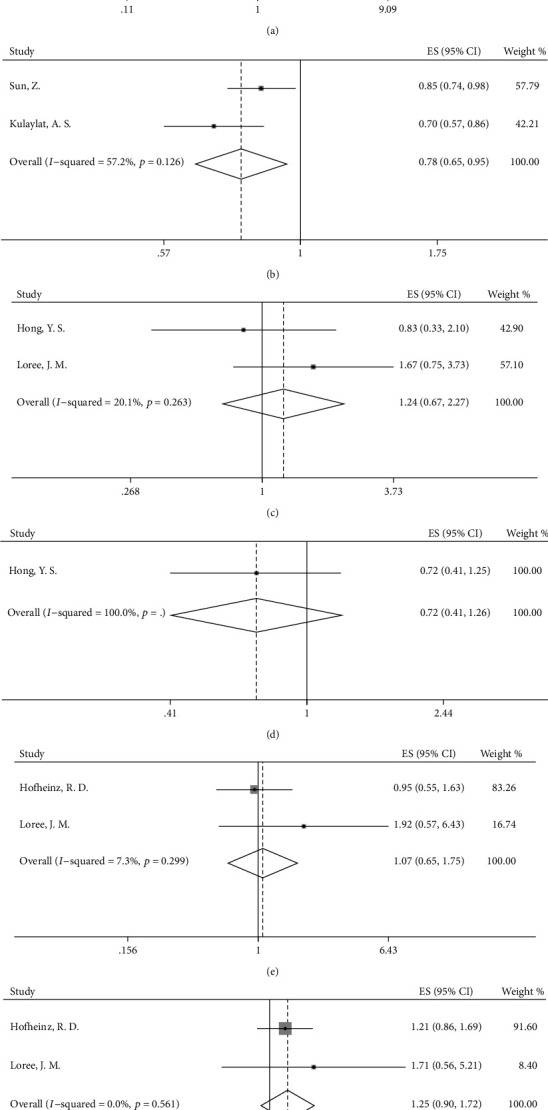
Forest plot based on overall survival and tumor stage. (a) OS in chemotherapy group versus observation group in patients with ypStage II; (b) OS in chemotherapy group versus observation group in patients with ypStage III; (c) OS in OX/FU-based group versus FU-based group in patients with ypStage II; (d) OS in OX/FU-based group versus FU-based group in patients with ypStage III; (e) OS in OX/FU-based group versus FU-based group in patients with clinical stage II; (f) OS in OX/FU-based group versus FU-based group in patients with clinical stage III.

Moreover, in a comparison between adjuvant chemotherapy and observation, subgroup analysis based on ypN showed that ypN^−^ patients who received adjuvant chemotherapy had better OS than those in the observation group (ypN^−^: HR = 0.66, 95%CI = 0.59–0.75, *p* < 0.001; ypN^+^: not applicable, owing to insufficient data). Further analysis based on ypStage showed that adjuvant chemotherapy contributed to better OS in both the ypStage II group and ypStage III (ypStage II: HR = 0.73, 95%CI = 0.60–0.88, *p* = 0.001, Figure 6(a); ypStage III: HR = 0.78, 95%CI = 0.65–0.95, *p* = 0.011, Figure 6(b)), but the data were insufficient for subgroup analysis in patients with ypStage 0 and ypStage I. In contrast, in the comparison between the OX/FU-based group and FU-based group, no OS benefit was observed in patients with either the ypN0 group or ypN+ group (ypN0: HR = 1.26, 95%CI = 0.68–2.36, *p* = 0.466; ypN1: HR = 1.05, 95%CI = 0.54–2.05; ypN2: HR = 0.42, 95%CI = 0.18–0.97, *p* = 0.042), and similar results were also found in further analysis based on ypStage (ypStage II: HR = 1.24, 95%CI = 0.67–2.27, *p* = 0.491, Figure 6(c); ypStage III: HR = 0.72, 95%CI = 0.41–1.26, Figure 6(d)). Similarly, no OS differences were observed in both clinical stage II and III subpopulations (clinical stage II: HR = 1.07, 95%CI = 0.65–1.75, *p* = 0.793, Figure 6(e); clinical stage III: HR = 1.25, 95%CI = 0.90–1.72, *p* = 0.183, Figure 6(f)).

In the chemotherapy group versus the observation group, nRCTs indicated a significant difference in OS, whereas RCTs showed a limited clinical benefit in terms of OS after adjuvant chemotherapy, and the results were not statistically significant (HR = 0.73, 95%CI = 0.67–0.79, *p* < 0.001; HR = 0.95, 95%CI = 0.82–1.09, *p* = 0.437). In OX/FU-based chemotherapy versus FU-based chemotherapy, there was no benefit in either RCTs or nRCTs when oxaliplatin was added (HR = 1.02, 95%CI = 0.85–1.22, *p* = 0.863; HR = 1.67, 95%CI = 0.75–3.72, *p* = 0.210). The details are shown in Table 2.

### 3.5. Toxicity

**Figure 7:**
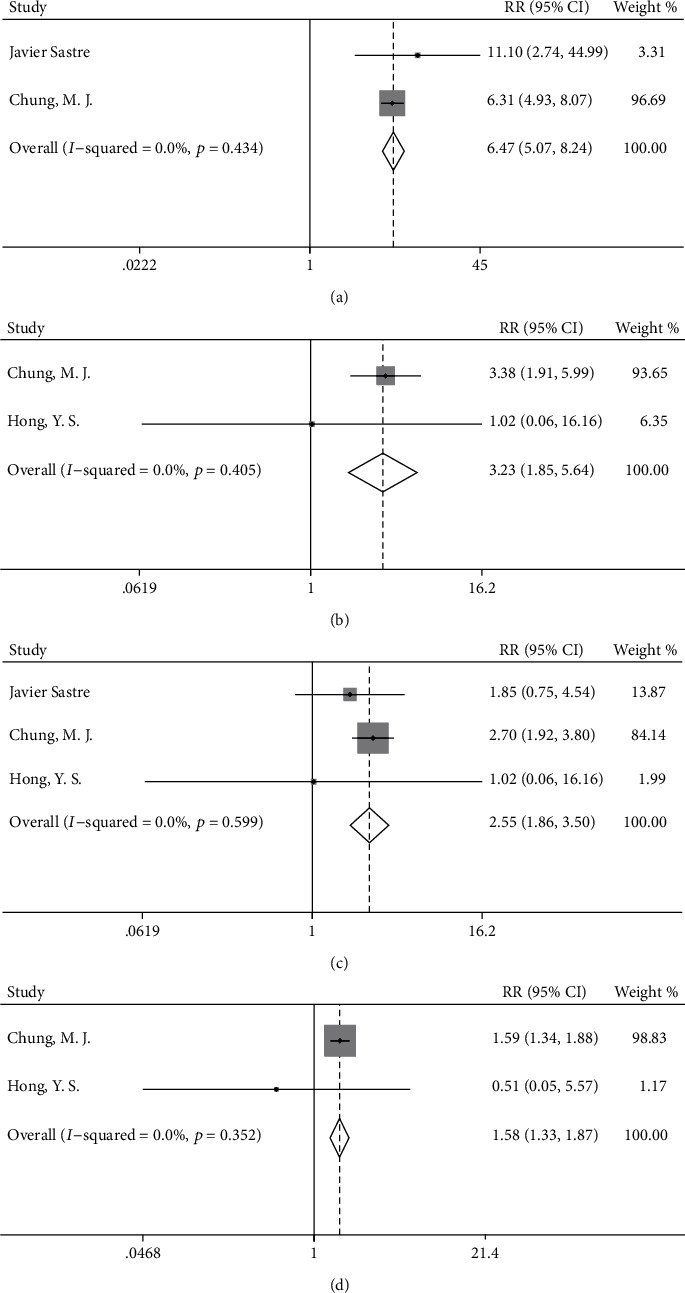
Forest plots for the incidence of (a) neuropathy, (b) allergic reaction, (c) vomiting, and (d) nausea in the oxaliplatin/fluorouracil-based group compared with fluorouracil-based group.

Our overall analyses demonstrated that the OX/FU-based group showed a significantly greater incidence of neuropathy (RR = 6.47, 95%CI = 5.07–8.24; *p* < 0.001, Figure 7(a)), allergic reaction (RR = 3.23, 95%CI = 1.85–5.64; *p* < 0.001, Figure 7(b)), vomiting (RR = 2.55, 95%CI = 1.86–3.50; *p* < 0.001, Figure 7(c)), and nausea (RR = 1.58, 95%CI = 1.33–1.87; *p* < 0.001, Figure 7(d)) than the FU-based group. In the OX/FU-based group versus the FU-based group, the incidence rates of neuropathy, allergic reaction, vomiting, and nausea were 23.5% (95%CI = 21.6% − 25.4%) vs. 3.6% (95%CI = 2.8% − 4.5%), 4.6% (95%CI=3.4%−5.9%) vs. 1.4% (95%CI=0.7%−2.1%), 11.1% (95%CI=9.3%−12.9%) vs. 4.3% (95%CI=3.1%−5.5%), and 25.1% (95%C=22.5%−27.7%) vs. 16.0% (95%CI=13.8%−18.2%), respectively. In the chemotherapy group versus the observation group, toxicity occurred only in patients receiving adjuvant chemotherapy, and we calculated the incidence rate of grade 3–4 toxicity. The rates of diarrhea, neuropathy, nausea, hand-foot syndrome, and vomiting were 17.0% (95%CI = 11.2% − 22.7%), 5.5% (95%CI = 2.0% − 8.9%), 5.5% (95%CI = 2.0% − 8.9%), 3.6% (95%CI = 0.8% − 6.5%), and 3.6% (95%CI = 0.8% − 6.5%), respectively. The detailed results of subgroup analyses of toxicity are shown in Figure 7.

### 3.6. Compliance

**Figure 8:**
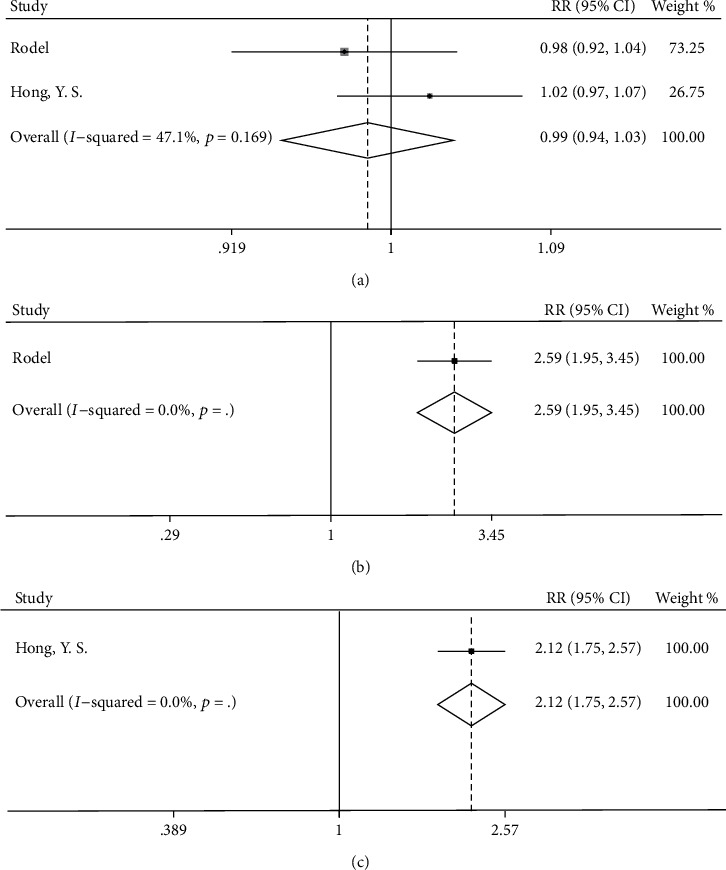
Forest plot for compliance in (a) the completion rate of the planned cycles, (b) the rate of dose reduction of patients, and (c) the rate of cycles with reduced doses in oxaliplatin/fluorouracil-based group versus fluorouracil-based group.

For the OX/FU-based group and FU-based group, only two studies reported compliance. According to our meta-analysis results, the compliance with OX/FU-based chemotherapy was comparable to that with FU-based chemotherapy, with low heterogeneity (RR = 0.99, 95%CI = 0.94–1.03, *p* = 0.590, Figure 8(a)). Only one study reported that dose reduction was more common in the OX/FU-based group than in the FU-based group (135 of 445, 30% vs. 55 of 470, 12%; RR = 2.59, 95%CI = 1.95–3.45, Figure 8(b)) [11]. Moreover, only one study indicated that cycles with reduced doses were more common in the OX/FU-based group than in the FU-based group (430 of 1153, 37.3% vs. 102 of 580, 17.6%; RR = 2.12, 95%CI = 1.75–2.57, Figure 8(c)) [12].

## 4. Discussion

In this analysis, we included 20 studies on 30662 patients. In the chemotherapy group versus the observation group, adjuvant treatment was found to be beneficial in terms of OS and DFS. Subgroup analysis based on neoadjuvant treatment strategy revealed similar results, in which the adjuvant chemotherapy group showed higher OS and DFS than those in the observation group in patients treated with preoperative long-course chemoradiation. In addition, the results showed an increase in OS in either ypStage II or ypStage III in the chemotherapy group versus the observation group. In the OX/FU-based group, compared with the FU-based group, the addition of oxaliplatin did not contribute to OS or DFS. The results were also demonstrated in the subgroup analysis of patients treated with neoadjuvant long-course chemoradiation, and there were no significant survival benefits in the clinical stage II, clinical stage III, ypStage II, and ypStage III groups. However, oxaliplatin clearly increased the incidence of neuropathy, allergic reaction, vomiting, and nausea. Regarding compliance in completing all adjuvant chemotherapy cycles, there was no significant difference in the OX/FU-based group versus the FU-based group. However, the OX/FU-based group had a much higher rate of dose reduction than the FU-based group.

It has been reported that patients with ypStage III can benefit from adjuvant chemotherapy [5, 6], which is in agreement with the present results. Regarding patients with ypStage II who received neoadjuvant treatment, one study has shown no increase in OS or DFS [29], whereas other studies have indicated that patients with ypStage II benefit from adjuvant chemotherapy [5, 6]. Present pooled analysis showed that adjuvant chemotherapy improved OS in ypStage II patients undergoing neoadjuvant treatment and surgery. Several potential explanations may account for the survival benefits from adjuvant chemotherapy in ypStage II patients. First, neoadjuvant chemoradiation may lead to downstaging, and a portion of ypStage II patients might be pN^+^ before receiving neoadjuvant treatment. In addition, patients with ypStage II mainly had T3–T4 stage cancer, which might infiltrate deeper before neoadjuvant treatment was received. Therefore, the adjuvant chemotherapy regimens guided by ypTNM stage after neoadjuvant chemoradiation may be different from those administered according to pTNM stage without neoadjuvant chemoradiation. Therefore, these reasons may plausibly explain the improved survival in ypStage II patients, which was different from that in pStage II patients. Studies that explore the efficacy of adjuvant chemotherapy following neoadjuvant treatment according to ypStage are needed for further validation.

Oxaliplatin-based adjuvant chemotherapy was provided for patients with LARC after neoadjuvant treatment and surgery. However, in our analysis, adding oxaliplatin to adjuvant chemotherapy did not confer a benefit in either OS or DFS. This result might have been due to the toxicity of oxaliplatin causing a dose reduction-a factor that would negatively affect survival. In some studies [4, 11, 15, 29], oxaliplatin was added to neoadjuvant treatment in the OX/FU-based group. The toxicity of oxaliplatin had cumulative effects, and using oxaliplatin-based adjuvant chemotherapy after oxaliplatin-based neoadjuvant treatment increased the toxicity, thereby affecting the efficacy of adjuvant chemotherapy [11]. Our results showed that oxaliplatin could lead to toxicity, which was clearly reflected by neuropathy, acute reaction, vomiting, and nausea. These side effects may decrease patient compliance. In the ADORE trial, cycles with reduced doses were more frequent in the OX/FU-based group than in the FU-based group [12]. Our meta-analysis indicated no significant difference in compliance in the completion rate of the planned cycles between the two groups, but dose reduction decreased the efficacy of adjuvant chemotherapy. Therefore, the decision to add oxaliplatin in adjuvant chemotherapy should account for the balance between efficacy and toxicity. Finally, a study has found that adding oxaliplatin is not associated with OS or DFS in patients ≥73 years of age [37]. Therefore, in this study, some patients were above 73 years old, thus, possibly affecting the final results. However, we did not discuss this possibility further because of a lack of data.

In clinical practice, evaluating the effects of neoadjuvant treatment strategy on the survival benefits of adjuvant treatment in patients with LARC is meaningful. Given that most of the included studies used neoadjuvant long-course chemoradiation, we further evaluated the survival benefits of adjuvant chemotherapy in the setting of neoadjuvant long-course chemoradiation. The results showed similar results to those of the overall analysis, indicating that adjuvant chemotherapy, compared with observation, contributes to survival benefit, and that adding oxaliplatin to FU-based adjuvant chemotherapy does not contribute to survival benefits beyond those conferred by FU-based adjuvant chemotherapy. In terms of neoadjuvant chemotherapy strategy, further subgroup analyses were conducted to evaluate the effects of neoadjuvant long-course chemoradiation with preoperative FU-based chemotherapy or neoadjuvant long-course chemoradiation with preoperative OX/FU-based chemotherapy on survival outcomes. Similar results were observed, thus, demonstrating the reliability and accuracy of our results. However, subgroup analysis based on preoperative radiotherapy strategy (short-course versus long-course radiotherapy) could not be conducted, owing to an insufficient number of studies. Thus, future large-scale, prospective clinical studies are needed to explore the influence of neoadjuvant treatment strategy, including preoperative short-course radiotherapy, preoperative long-course radiotherapy, and long-course chemoradiation, on the relationship between adjuvant chemotherapy and survival benefits. Such an understanding may lead to more effective and suitable clinical treatment strategies for patients with LARC.

There are several limitations to the current research. First, this was a retrospective analysis, and the potential for confounding on the basis of patient selection could not be eliminated. Second, we were unable to obtain the personal details of patients, although this information might have enabled better control of the confounding factors. Third, the heterogeneity in the study that could not be eliminated by subgroup analysis might have affected external authenticity to some extent. Moreover, some patients may have better pathological responses to neoadjuvant treatment, thus, achieving ypStage1 or even ypStage 0 after neoadjuvant treatment; however, the survival benefit of adjuvant chemotherapy still remains unclear for these patients in clinical practice. Unfortunately, we could not perform further analysis because the data for patients with ypStage 0 or I after neoadjuvant treatment were insufficient. In addition, we were unable to perform more subgroup analyses because of the insufficient number of studies. Finally, the recent guidelines for adjuvant chemotherapy regimens are mainly based on pStage. Differences exist between ypStage and pStage, and, thus, chemotherapy guided by ypStage might require further exploration. Future studies are needed to further investigate and resolve these problems.

## 5. Conclusions

Compared with observation, adjuvant chemotherapy was found to prolong OS in patients with LARC after neoadjuvant (chemo) radiotherapy and curative surgery. However, no survival benefit was observed with the addition of oxaliplatin to FU-based adjuvant chemotherapy compared with FU-based adjuvant chemotherapy.

## Figures and Tables

**Table 1:** The baseline characteristics and study quality of included studies.

Study	Country and year	Study design	No. of patients (M/F)	Arm	Age mean ± SD/median (range)	Follow-up mean ± SD/median (range)	T category (0-2/3/4/NR)	N category (N-/N+/NR)	TNM stage (0-I/II/III/IV/NR)	Type of neoadjuvant chemotherapy	Type of adjuvant chemotherapy	NOS
Javier Sastre	Spain 2016	nRCT	87	OX	69 (35-86)	NR	ypT3-4: 50	yp: -/50/-	NR	5.4 Gy boost + 45 Gy/25F, CAP+5-FU or UTF	FOLFOX or XELOX	7
			(50/37)	FL		NR	ypT0-2: 37	yp: 37/-/-	NR		CAP or 5 − FU + LV or raltitrexed	
Rodel	German 2015	RCT	1236	OX	Median: 64	50 (38–61) m	yp: 316/260/17/3	yp: 416/175/5	yp:252/154/154/20/16	50.4Gy/28F, FU+ oxaliplatin	FU + LV+ oxaliplatin	7
			(874/362)	FL	Median: 63		yp: 310/278/26/1	yp: 423/191/1	yp:257/148/169/35/6	50.4 Gy/28F, FU	FU	
Hong, Y. S.	Korea	RCT	321	OX	54 (25-79)	74.1 (56.2-88.0) m	yp: 24/133/3/-	yp: 58/102/-	yp: -/58/102/-/-	50Gy, FU ± LV or CAP or tegafur/uracil	FU + LV+ oxaliplatin	8
	2019		(234/87)	FL	55 (27-81)		yp: 24/131/6/-	yp: 65/96/-	yp: -/65/96/-/-		FU + LV	
Chung, M. J.^∗^	Korea	nRCT	1442	OX	NR		ypT0-2: 10, ypT3-4: 58	yp: 13/55/-	NR	45 Gy/25F + 5.4 Gy/3F, 5 − FU + LV + CAP	5 − FU + LV + oxaliplatin	8
	2019		(975/467)	FL	NR	48.8 (6.5-141.0) m	ypT0-2: 37, ypT3-4: 167	yp: 37/167/-	NR		5 − FU + LV	
				OB	NR		ypT0-2: 96, ypT3-4: 85	yp: 133/48/-	NR		/	
Loree, J. M.	Canada	nRCT	485	OX	61 (52-68)	Median: 5.1y	NR	NR	c: 5/136/201/-/15	45 to 54 Gy, 5-FU or CAP or FOLFOX or IXO	FOLFOX or CAPOX	7
	2018		(343/142)	FL		Median: 3.64y	NR	NR			5-FU or CAP	
				OB	64 (56-72)	Median: 4.4y	NR	NR	c: 3/44/72/-/9		/	
Garlipp, B.	German	nRCT	1497	OX	65 (29.0–88.0)		yp: 71/82/7/-	yp: 86/74/-	NR		Oxaliplatin +5-FU or FA or CAP	8
	2016		(1006/490)	FL	67 (26.0–85.0)	Median: 38 m	yp: 356/383/29/-	yp: 522/246/-	NR	5FU-only-based CRT	5-FU or FA or CAP	
				OB	68.5 (23.0–89.0)		yp: 283/268/17/1	yp: 435/134/-	NR		/	
Peng, J. H.	China	nRCT	105	CT	51.3 ± 11.4	49 (4–89) m	c: 4/54/25/-	c: 27/56/-	c: -/27/56/-/-	46–50Gy, XELOX	XELOX	7
	2018		(70/35)	OB	58.9 ± 11.6		c: 0/14/8/-	c: 8/14/-	c: -/8/14/-/-		/	
Glynne-Jones, R.	UK	RCT	113	CT	59.0 (55.0–66.0)	Median: 44.8 m	yp: 25/27/2/-	yp: 44/10/-	NR	5FU-based CRT	XELOX	5
	2014		(30/83)	OB	58.0 (52.0–65.0)	Median: 44.8 m	yp: 17/38/4/-	yp: 31/28/-	NR		/	
You, K. Y.	China	nRCT	160	CT	54 (15–80)	Median: 47 m	yp: 65/37/10/3	c: 41/74/-	c: -/41/74/-/-	46Gy/23F, FOLFOX-6 or XELOX	FOLFOX or XELOX or CAP	6
	2014		(119/41)	OB	62 (39–77)	Median: 41 m	yp: 26/16/3/-	c: 20/25/-	c: -/20/25/-/-		/	
Sun, Z.	USA	nRCT	12696	CT	56.0 (48–64)	NR	NR	NR	yp: -/1586/2437/-/-	Chemoradiation	NR	6
	2017		(7853/4843)	OB	60.0 (51–69)	NR	NR	NR	yp: -/4401/4272/-/-		NR	
Sainato, A.	Italy 2014	RCT	634 (421/213)	CT	Mean: 60.5	Median: 63.7 m	ypT0-2: 150, ypT3-4: 143, ypTx: 3	c: 168/112/44	NR	45 Gy/28F, 5-FU + FA	5-FU + FA	8
				OB	Mean: 60.6	Median: 63.7 m	ypT0-2: 169, ypT3-4: 120, ypTx: 5	c: 194/76/40	NR		/	
Kulaylat, A. S.	USA	nRCT	8344	CT	NR	NR	NR	yp: 2645/1527/-	c: -/1823/2349/-/-	Chemoradiation	NR	7
	2017		(5212/3132)	OB	NR	NR	NR	yp: 3063/1109/-	c: -/1869/2303/-/-		NR	
Kuan, F. C.	China	nRCT	259	CT	56.67 ± 13.04	38 (26-56) w	c: 11/93/9/1	c: 33/81/-	c: -/33/81/-/-	40-60Gy, 5-FU/LV, tegafur or CAP	NR	6
	2017		(164/95)	OB	61.88 ± 11.31	36 (23-56) w	c: 21/114/10/-	c: 52/91/2	c: -/54/91/-/-		NR	
Kiran, R. P.	USA	nRCT	128	CT	55.6 ± 11.8	51.2 (25.8–68.1) m	yp: 29/28/1/-	NR	yp: 28/30/-/-/-	50.4 Gy, 5-FU or 5 − FU + LV	NR	8
	2012		(90/38)	OB	59.4 ± 12.1	54.1 (34.7–78.2) m	yp: 51/19/0/-	NR	yp: 48/22/-/-/-		NR	
Jung, K. U.	Korea	nRCT	476	CT	54.0 (46.5-62.5)	Median: 48.4 m	yp: 217/206/18/-	yp: 301/140/-	yp: 182/118/141/-/-	44 Gy/22F, 5-FU or Xeloda or FU + LV	5-FU	8
	2015		(312/164)	OB	64.0 (47.0–72.0)	Median: 42.1 m	yp: 17/17/1/-	yp: 23/12/-	yp: 17/6/12/-/-		/	
Geva, R.	Israel	nRCT	52	CT	60.9 ± 11.9	68.4 ± 43.4 m	c: 1/31/-/3	c: 23/9/3	NR	45 or 50.4Gy, 5-FU or CAP	NR	8
	2014		(32/20)	OB	68.7 ± 10.8	49.4 ± 38.9 m	c: 2/14/-/1	c: 12/3/2	NR	50.4Gy, 5-FU or CAP	NR	
Breugom, A. J.	Netherlands	RCT	437 (270/167)	CT	Median: 61.13 ± 8.94	5.0 (0.02–13.12) y	NR	NR	yp: -/39/177/-/-	25Gy/5F or 45–50 Gy/25F, 5-FU	5-FU or LV	8
	2015			OB	Median: 61.08 ± 9.13		NR	NR	yp: -/32/189/-/-		/	
Bosset, J. F.	France	RCT	1011	CT	NR	10·4 (7·8–13·1) y	c: -/454/52/-	ypN+: 140	NR	45 Gy/25F; 45 Gy/25F, FU + LV	FU + LV	8
	2014		(739/272)	OB	NR		c: -/456/49/-	ypN+: 154	NR		/	
Ahn, D. H.	USA	nRCT	110	CT	54.3 (27-76)	NR	yp: 68/-/-/3	p: 37/28/6	NR	Chemoradiation	5-FU or CAP or FOLFOX	5
	2017		(68/42)	OB	62 (21-79)	NR	yp: 38/-/-/1	p: 29/7/3	NR		/	
Hofheinz, R. D.	German	RCT	1069	OX	NR	Median: 31 m	NR	NR	NR	45-50.4 Gy, CAP + OX	CAP + OX	6
	2017			FL	NR	Median: 31 m	NR	NR	NR	45-50.4 Gy, CAP	CAP	

^∗^Patient characteristics based on T category and N category were after propensity-score matching. RCT: randomized controlled trial; nRCT: nonrandomized controlled trial; M/F: male/female; OX: oxaliplatin-based group; FL: fluorouracil-based group; OB: observation group; CT: chemotherapy group; m: month; y: year; w: week; c: clinical stage; yp: pathologic stage after receiving neoadjuvant chemotherapy; p: pathologic stage; FU: fluorouracil; UTF: uracil + tegafur + fluorouracil; LV: leucovorin; CAP: capecitabine; FA: folinic acid; FOLFOX: fluorouracil + leucovorin + oxaliplatin; XELOX: capecitabine + oxaliplatin; CAPOX: capecitabine + oxaliplatin; IXO: irinotecan + capecitabine + oxaliplatin; “/”: there is no relevant data; NR: not reported; NOS: Newcastle–Ottawa Scale criteria.

**Table 2:** Subgroup analysis of overall survival and disease-free survival.

	Subgroup	OS					DFS				
		No. of study	HR	95% CI	*P* for HR	Heterogeneity (*P*, *I*^2^)	No. of study	HR	95% CI	*P* for HR	Heterogeneity (*P*, *I*^2^)
Oxaliplatin-based group versus fluorouracil-based group	*Study design*										
	RCT	3	1.02	0.85-1.22	0.863	0.199, 38.0%	/	/	/	/	/
	nRCT	1	1.67	0.75-3.72	0.210	/	/	/	/	/	/
	*Neoadjuvant treatment regimen*										
	Chemoradiation	4	1.04	0.87-1.24	0.656	0.201, 35.2%	6	0.99	0.76-1.27	0.906	0.016, 64.0%
	RT + FU	/	/	/	/	/	3	1.10	0.57-2.10	0.782	0.007, 79.6%
	*ypStage*										
	ypStage II	2	1.24	0.68-2.27	0.491	0.263, 20.1%	3	0.82	0.59-1.13	0.225	0.262, 25.4%
	ypStage III	1	0.72	0.41-1.26	0.248	/	2	0.74	0.50-1.11	0.142	0.155, 50.5%
	*Clinical stage*										
	Clinical stage II	2	1.07	0.65-1.75	0.793	0.299, 7.3%	1	0.95	0.59-1.52	0.831	/
	Clinical stage III	2	1.25	0.90-1.72	0.183	0.561, 0.0%	2	1.09	0.84-1.41	0.533	0.275, 16.0%
	*ypN*										
	ypN0	2	1.26	0.68-2.36	0.466	0.275, 16.1%	3	0.83	0.64-1.08	0.16	0.316, 13.2%
	ypN1	2	1.05	0.54-2.05	0.888	0.474, 0.0%	2	1.05	0.52-2.10	0.897	0.411, 0.0%
	ypN2	1	0.42	0.18-0.97	0.042	/	2	0.75	0.33-1.70	0.492	0.070, 69.5%

Chemotherapy group versus observation group	*Study design*										
	RCT	4	0.95	0.82-1.09	0.437	0.836, 0.0%	/	/	/	/	/
	nRCT	11	0.73	0.67-0.79	<0.001	0.038, 47.9%	/	/	/	/	/
	*Neoadjuvant treatment regimen*										
	Chemoradiation	13	0.73	0.61-0.89	0.001	0.013, 52.8%	10	0.69	0.51-0.94	0.018	0.002, 65.8%
	RT + OX	2	0.49	0.20-1.24	0.133	0.396, 0.0%	2	0.41	0.21-0.78	0.007	0.437, 0.0%
	*ypStage*										
	ypStage II	3	0.73	0.60-0.88	0.001	0.481, 0.0%	2	0.57	0.16-2.11	0.401	0.113, 60.2%
	ypStage III	2	0.78	0.65-0.95	0.011	0.126, 57.2%	/	/	/	/	/
	*ypN*										
	ypN0	7	0.66	0.59-0.75	<0.001	0.839, 0.0%	/	/	/	/	/

RCT: randomized controlled trial; nRCT: nonrandomized controlled trial; RT: radiotherapy; OX: oxaliplatin-based chemotherapy; FU: fluorouracil-based chemotherapy; OS: overall survival; DFS: disease-free survival; HR: hazard ratio; CI: confidence interval; *I*^2^: degree of heterogeneity; ypStage: pathologic stage after receiving neoadjuvant chemotherapy; “/”: there is no relevant data.

## Data Availability

The research data used to support the findings of this study were supplied by Zhenning Wang under license and so cannot be made freely available. Requests for access to these data should be made to Zhenning Wang, E-mail: josieon826@sina.cn.

## Supplementary Materials

Supplementary Materials Supplementary File 1: PRISMA checklist. Supplementary File 2: detailed search strategy and result for each database.
