# Supplementary material for: Postoperative Adjuvant Treatment Strategy for Locally Advanced Rectal Cancer after Neoadjuvant Treatment
Source: Biomed Res Int. 2021 Mar 28;2021:8852699. doi: 10.1155/2021/8852699 (PMC8319733; doi:10.1155/2021/8852699)
Supplement: Supplementary Materials — Supplementary File 1: PRISMA checklist. Supplementary File 2: detailed search strategy and result for each database. [file 8852699.f1.zip › 8852699.f1/Supplementary File 2.docx]

**Literature Search (up to December, 2019)**

A systematical and comprehensive literature search was performed in following databases: PubMed and Embase databases.

**Detailed search strategy and result for each database:**

**1. PubMed: 977 studies**

Search in All Fields:

(neoadjuvant chemotherapy OR neoadjuvant radiotherapy OR neoadjuvant chemoradiotherapy OR neoadjuvant treatment OR neoadjuvant treatments OR neoadjuvant therapy OR neoadjuvant therapies OR preoperative chemotherapy OR preoperative radiotherapy OR preoperative chemoradiotherapy OR preoperative treatment OR preoperative treatments OR preoperative therapy OR preoperative therapies OR pre-operative chemotherapy OR pre-operative radiotherapy OR pre-operative chemoradiotherapy OR pre-operative treatment OR pre-operative treatments OR pre-operative therapy OR pre-operative therapies) AND (rectal cancer OR colorectal cancer) AND oxaliplatin

**2. Embase: 3764 studies**

Search in All Fields:

(neoadjuvant chemotherapy OR neoadjuvant radiotherapy OR neoadjuvant chemoradiotherapy OR neoadjuvant treatment OR neoadjuvant treatments OR neoadjuvant therapy OR neoadjuvant therapies OR preoperative chemotherapy OR preoperative radiotherapy OR preoperative chemoradiotherapy OR preoperative treatment OR preoperative treatments OR preoperative therapy OR preoperative therapies OR pre-operative chemotherapy OR pre-operative radiotherapy OR pre-operative chemoradiotherapy OR pre-operative treatment OR pre-operative treatments OR pre-operative therapy OR pre-operative therapies) AND (rectal cancer OR colorectal cancer) AND oxaliplatin
